# High-Flux Solvent-Resistant Reverse Osmosis Membrane Enabled by D-glucamine Surface Modification

**DOI:** 10.3390/membranes16050171

**Published:** 2026-05-06

**Authors:** Bing Wang, Weijia Song, Yuqi Sun, Enlin Wang, Can Li, Baowei Su

**Affiliations:** 1 Key Laboratory of Marine Chemistry Theory and Technology (Ocean University of China), Ministry of Education/College of Chemistry & Chemical Engineering, Ocean University of China, 238 Songling Road, Qingdao 266100, China; 2 Singapore Membrane Technology Centre, Nanyang Environment and Water Research Institute, Nanyang Technological University, 1 Cleantech Loop, Singapore 637141, Singapore

**Keywords:** organic solvent reverse osmosis (OSRO), surface modification, D-glucamine, organic solvent separation, high flux

## Abstract

Organic solvent reverse osmosis (OSRO) is an emerging membrane technology for low-energy separation of organic mixtures, yet developing OSRO membranes with both high permeance and robust stability remains challenging. Herein, we present a surface modification strategy using a D-glucamine/ethanol solution to tailor the physicochemical properties of a crosslinked polyimide-supported polyamide OSRO membrane. D-glucamine, as an amino sugar alcohol compound contains a primary amino group and multiple hydroxyl groups, endowing it with specific chemical reactivity and potential for interface modification. The optimized OSRO membrane exhibited a significantly decreased water contact angle from 52.6° of the control membrane to 36.6°, indicating substantially enhanced surface hydrophilicity. The optimized membrane (TFC-D-0.2) achieves a high water permeance of 12.84 LMH/MPa with a NaCl rejection of 98.25% and demonstrates excellent operational stability and pressure resistance (2.5~4.0 MPa). The membrane also shows good tolerance to most organic solvents, maintaining >96.5% NaCl rejection after 30 days of immersion in all tested solvents except acetone. In concentrating ethyl cinnamate/ethanol mixtures over 50 h, the membrane delivers stable performance with an ethanol permeance of ~2.5 L m^−2^ h^−1^ MPa^−1^ and a solute rejection of >88%. This work provides an effective surface modification strategy for developing high-performance OSRO membranes, holding promise for green separation processes in fine chemical industries.

## 1. Introduction

The separation of industrial chemicals predominantly involves organic liquids, particularly low-molecular-weight ones (<200 Da). Currently, organic solvent separation relies heavily on energy-intensive phase change methods, such as evaporation and distillation. According to statistics, the associated carbon emissions and energy consumption account for more than 10% of the global total [1]. Notably, phase change operations, such as distillation, consume approximately 80% of the total energy used in chemical separation [2]. Meanwhile, these separation processes would usually cause the emissions of vast high volatile organic compounds (VOCs), which are harmful to human health and the environment. Therefore, green low-energy separation technologies are urgently needed.

Membrane technologies have long been considered as a highly efficient and promising technology for separating liquid organic solvent mixtures. Currently, there are three membrane technologies that can be used for separating organic liquid mixtures, including organic solvent nanofiltration (OSN), pervaporation (PV), and organic solvent reverse osmosis (OSRO). Among these three, OSN membranes are suitable for separating solutes with a molecular weight of 200–1000 Da. In contrast, PV and OSRO target systems with molecular weights below 200 Da or differences in molecular size of 1–3 Å. PV involves higher energy consumption than OSRO due to phase changes [3]. OSRO, by comparison, is a phase-change-free process which consumes only 1–10% of the energy required for PV and its energy consumption is the lowest among the three membrane technologies [4]. Meanwhile, it can operate at room temperature, making it suitable for heat-sensitive substances [4]. Therefore, OSRO technology has significant research and application value. This potential is particularly promising in the pharmaceutical and fine chemical industries, where the recovery of high-value products, the concentration of thermally unstable intermediates, and the purification of active pharmaceutical ingredients under mild conditions are critical requirements.

The concept of OSRO membranes originated in 1964, when Sourirajan [5] developed cellulose acetate membranes for separating binary organic mixtures, such as xylene/ethanol. However, there was little development in the following decades, primarily due to a lack of suitable solvent-resistant materials. In recent years, significant progress has been made on OSRO membrane fabrication. In particular, interfacial polymerization (IP), a widely used technique for fabricating reverse osmosis (RO), nanofiltration (NF) and OSN membranes, was successfully employed for developing thin-film composite (TFC) OSRO membranes in 2020 [6].

Interfacial polymerization could form thin, defect-free selective layers with high rejection, high flux and structural integrity, and has been proven to be one of the most effective methods for the fabrication of TFC OSRO membranes. The separation layer of most TFC OSRO membranes is made of polyamide (PA) via interfacial polymerization, where the monomer in the aqueous phase is usually m-phenylenediamine (MPD), while the monomer in the organic phase is typically trimesoyl chloride (TMC). Professor Matsuyama’s group has conducted extensive research in this area. In 2020, they successfully fabricated high-performance OSRO membranes via IP using an ultrafiltration polyketone membrane with excellent solvent resistance as the substrate [6]. The resulting OSRO membrane exhibited an NaCl rejection of over 97% and a water permeance of 5 L m^−2^ h^−1^ MPa^−1^ (shorten as LMH/MPa). Meanwhile, the OSRO membrane achieved solute/solvent separation factors of 8.4, 11.1, 14.9, and 38.0 for 10 wt% methanol solutions containing toluene, pentane, hexane, and heptane, respectively, at a pressure of 3.0 MPa, with a corresponding methanol permeance of approximately 1.7 LMH/MPa. Subsequently, various strategies have been applied to enhance the separation performance of the polyketone-based OSRO membranes, including simple heat treatment [7], controlling fabrication temperature conditions [8,9], altering the monomers in the aqueous phase [10,11], surface modification [12,13], and constructing intermediate layers [14]. Till now, most OSRO membranes are hydrophilic and are for polar solvent separation, except some recent works by Professor Livingston’s group [15], Smith et al. [16] and Prof. Xu’s group [17] on hydrophobic membranes for liquid hydrocarbon separation. However, the current permselectivity of most hydrophilic OSRO membranes remains relatively low, which is a critical challenge hindering their development. This issue is particularly pronounced in PA-based TFC membranes, where achieving an optimal balance between a highly crosslinked, selective structure and sufficient surface hydrophilicity for high flux remains difficult. In this context, drawing inspiration from advanced membrane design strategies—such as the use of multifunctional monomers for interface engineering [18], precise regulation of membrane pore architecture [19,20], and the construction of hydrophilic-hydrophobic balanced surfaces [21]—offers a promising pathway to overcome these limitations. Therefore, there is an urgent need to develop effective strategies that enhance membrane permeance without compromising selectivity.

The surface properties of membranes are critical factors in determining their separation performance [22]. To enhance the overall performance of membranes, researchers typically use surface modification techniques to regulate the surface properties precisely without altering the macroscopic characteristics of the membrane as a whole [23,24]. Surface modification methods are primarily categorized as physical or chemical. The latter encompasses various techniques, such as chemical coupling, free-radical graft polymerization and atom transfer radical polymerization [23]. Notably, a simple and highly efficient surface modification approach involves utilizing the residual acyl chloride groups remaining on the membrane surface after IP to react with modifying monomers for grafting, thereby imparting specific functionalities to the membrane surface [22,25,26,27].

Amine compounds are excellent surface modification monomers. Their reactive amino groups can react with residual acyl chloride groups on the membrane surface, enabling the grafting of target functional groups onto the membrane surface and thereby imparting specific surface properties to the membrane. Typical amine compounds used for surface grafting include tris(2-aminoethyl)amine (TAEA) [28], monoethanolamine (MEA) [29], diethylenetriamine (DETA) [30], diethanolamine (DEA) and bis(2-methoxyethyl)amine (BMEA) [31]. D-glucamine as an amino sugar alcohol features a primary amino group (-NH_2_) that could undergo amidation with residual acyl chloride groups on the pristine PA membrane surface. We consider that D-glucamine can be used as an ideal candidate for the surface modification of nascent PA OSRO membrane surface, due to its unique combination of a reactive primary amine for covalent attachment to the residual acyl chloride groups and a hydrophilic sugar alcohol backbone that can significantly enhance surface wettability. However, to the best of our knowledge, no study has reported the use of D-glucamine for this purpose so far.

To address the challenge of balancing permeance and stability in OSRO membranes, we report a facile surface modification strategy using D-glucamine to fabricate high-performance TFC OSRO membranes. D-glucamine is covalently grafted onto the nascent polyamide layer via amide bonds with residual acyl chloride groups, introducing abundant hydroxyl groups that significantly enhance surface hydrophilicity. Meanwhile, D-glucamine facilitates surface densification through covalent bonding and hydrogen-bond network formation, while ethanol promotes polymer chain rearrangement and selective layer thinning. The resulting membranes achieve an exceptional balance of high solvent permeance and robust stability. Systematic optimization reveals that the prepared membranes exhibit high permeability and excellent stability, showing great potential for fine chemical separation. Overall, this study provides a novel and effective design strategy for high-performance OSRO membranes.

## 2. Materials and Methods

### 2.1. Materials

Lenzing P84 fibers was supplied by HP Polymer GmbH (Lenzing, Austria). m-Phenylenediamine (MPD, A.R.), 1,3,5-benzenetricarbonyl trichloride (TMC, A.R.), D-glucamine (A.R.), vanillin (152 Da, A.R.), 7-hydroxycoumarin (162 Da, A.R.), acetylsalicylic acid (180 Da, A.R.), ibuprofen (206 Da, A.R.), ethyl cinnamate (176 Da, A.R.), and 2-hydroxy-4-methoxybenzophenone (228 Da, A.R.) were obtained from Shanghai Maclin Biochemical Technology Co., Ltd. (Shanghai, China). Methanol (MeOH), ethanol (EtOH), isopropyl alcohol (IPA), ethyl acetate (EA), acetonitrile (ACN), n-hexane, and N,N-dimethylformamide (DMF) (all A.R.) were purchased from Tianjin Fuyu Fine Chemical Co., Ltd. (Tianjin, China). 1,6-Hexanediamine (HDA), acetone, and sodium chloride (NaCl) (all A.R.) were obtained from Sinopharm Chemical Reagent Co., Ltd. (Shanghai, China).

### 2.2. Fabrication of the TFC OSRO Membranes

**Figure 1:**
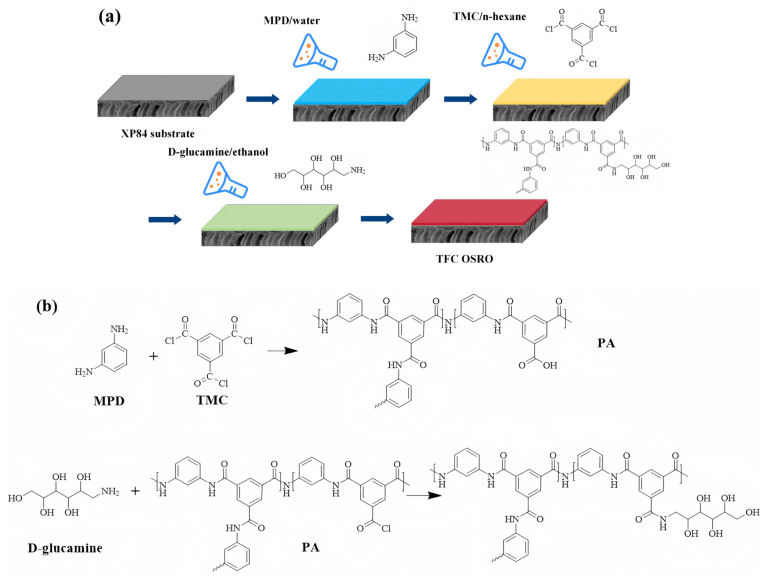
Preparation process (**a**) and reaction schemes (**b**) of the TFC OSRO membrane.

The PI substrate was prepared via the non-solvent induced phase separation method following previous work [32], and the TFC membrane was fabricated using IP. The specific procedure is shown in Figure 1.

Preparation of the PI support membrane: A casting solution was prepared by stirring 18 wt% P84 polymer powder and 1.0 wt% PEG-400 until completely dissolved in DMF. After standing for degassing and centrifugation, the solution was cast uniformly onto a non-woven fabric at 25 ± 2 °C and a relative humidity of 50 ± 5% using a casting knife. The cast film was air-evaporated for 10 s, then immersed in a water bath at 25 °C for phase inversion. After 10 min, the formed PI support membrane was removed and stored in deionized water. This PI support membrane was immersed in a 10.0 wt% HDA/IPA crosslinking agent at 60 °C for 30 min, then was subsequently stored in deionized water prior to the IP process.

Preparation of TFC OSRO membranes: First, the crosslinked PI support membrane was affixed flatly onto a plexiglass frame. After the membrane surface was air-dried, the aqueous phase monomer solution (2 wt% MPD) was rapidly poured onto the membrane. After 16 s, the solution was discarded, and any residual droplets were removed from the surface using a glass roller. The membrane was then air-dried for 60 s. Subsequently, the organic phase monomer solution (0.15 wt% TMC/n-hexane) was slowly and uniformly poured onto the membrane surface and allowed to react for 12 s to complete the IP reaction, yielding the benchmark OSRO membrane which is denoted as TFC. Afterward, D-glucamine/ethanol solutions of varying concentrations were poured onto the nascent PA membrane surface and permitted to react for a specific period to complete the surface modification. The modified OSRO membranes were then stored in deionized water for subsequent performance testing. For clarity, the modified OSRO membranes were denoted as TFC-D-*x*, where *x* represents the concentration (wt%) of the D-glucamine/ethanol solution used for surface modification. For instance, TFC-D-0.2 refers to the TFC OSRO membrane after modification with a D-glucamine concentration of 0.2 wt%, while TFC-D-0 denotes the TFC OSRO membrane after modification with pure ethanol. Different membranes are listed in Table 1 along with their preparation conditions.

### 2.3. Characterizations

The chemical structure and elemental composition of the membrane surface were analyzed using X-ray photoelectron spectroscopy (XPS, Thermo SCIENTIFIC ESCALAB Xi+, Thermo Fisher Scientific, Waltham, MA, USA). The surface and cross-sectional morphologies of the membranes were characterized using scanning electron microscopy (SEM, Zeiss GeminiSEM 360, CARL ZEISS, Oberkochen, Germany). The surface roughness of the membrane was measured using atomic force microscopy (AFM, OXFORD Cypher ES, Oxford Instruments, Santa Barbara, CA, USA). The hydrophilicity of the membrane surface was evaluated using a contact angle goniometer (CA, DSA100, Krüss, Hamburg, Germany).

### 2.4. Separation Performance

The separation performance of the fabricated OSRO membranes was evaluated using a laboratory-made cross-flow filtration apparatus with an effective membrane area of 28.26 cm^2^. The feed solution was an aqueous NaCl solution with a concentration of 2000 mg kg^−1^. Throughout the testing process, the temperature was maintained at 25 °C, and the operating pressure was set at 1.5 MPa. Prior to each measurement, the membrane was pre-pressurized at 1.7 MPa for at least 30 min to stabilize the flux. The permeance and rejection of the membranes were calculated using Equations (1) and (2), respectively.

(1) $$P=\frac{\Delta m}{\rho \cdot A\cdot \Delta t\cdot \Delta p}$$

(2) $$R=\left(1-\frac{C_{p}}{C_{f}}\right)\times 100\%$$

where *P* is the water permeance, L m^−2^ h^−1^ MPa^−1^ (shorten as LMH/MPa); Δ*m* is the mass of the permeate collected over a certain period, kg; *ρ* is the density of the permeate, kg L^−1^; Δ*t* is the testing time, h; Δ*p* is the trans-membrane pressure difference, MPa; *A* is the effective filtration area of the membrane, m^2^; *R* represents the rejection, -; *C_p_* and *C_f_* are the solute concentrations in the permeate and the feed solutions, respectively, mg kg^−1^. The concentration of the NaCl aqueous solution was determined by measuring its conductivity with a conductivity meter.

Furthermore, a range of small molecular solute/ethanol solutions (solute concentration of 100 mg kg^−1^) was used to evaluate the separation performance of the OSRO membranes in organic solvent systems. During testing, the temperature was maintained at 25 °C, the operating pressure was set at 3.0 MPa, and the membrane was pre-pressurized at 3.5 MPa for at least 30 min before each measurement. The permeance and solute rejection of the membrane were calculated using the above equations. The compositions of the permeate and feed solutions for the small molecular solute/ethanol systems were determined by measuring the solution absorbance using a UV spectrophotometer (UV-759).

### 2.5. Molecular Weight Cut-Off

The molecular weight cut-off (MWCO) of the OSRO membrane can be determined by testing its rejection for substances of different molecular weights. In this study, the rejection of the OSRO membrane for five small molecular substances in an ethanol solution: vanillin (152 Da), 7-hydroxycoumarin (162 Da), acetylsalicylic acid (180 Da), ibuprofen (206 Da), and 2-hydroxy-4-methoxybenzophenone (228 Da), were tested under an operating pressure of 3.0 MPa, with the solute concentration of each feed solution being fixed at 100 mg/kg. A curve illustrating the rejection as a function of molecular weight was plotted, from which the MWCO of the OSRO membrane was obtained. The chemical structures, molecular weights, and molecular size diameters of the five small-molecule compounds are shown in Table 2.

### 2.6. Solvent Resistance Evaluation

The solvent resistance of the TFC OSRO membranes was evaluated by immersing the membranes in six solvents—methanol, ethanol, isopropanol, acetone, n-hexane, and ethyl acetate—for 30 days. After immersion, the membranes were rinsed with deionized water, and their separation performance was tested using a 2000 mg kg^−1^ NaCl aqueous solution at 25 °C and 1.5 MPa to assess any changes.

### 2.7. Applications of the OSRO Membranes

To evaluate the application of the TFC OSRO membrane in the concentration of an ethyl cinnamate/ethanol solution, experiments were conducted using a laboratory-scale cross-flow filtration system. The optimized OSRO membrane was tested with a feed solution containing 85 mg kg^−1^ of ethyl cinnamate in ethanol at 25 °C and a transmembrane pressure of 3.0 MPa over a period of 10 h. During the experiment, the permeate was continuously removed to ensure effective concentration. Samples of both the permeate and the retentate were collected at intervals of 1 to 3 h.

## 3. Results and Discussion

### 3.1. Characterization of Membrane Surface Properties

The properties of the membrane surface were characterized using a range of analytical methods, including XPS, SEM, AFM, and WCA.

#### 3.1.1. Surface Chemistry of OSRO Membranes

**Figure 2:**
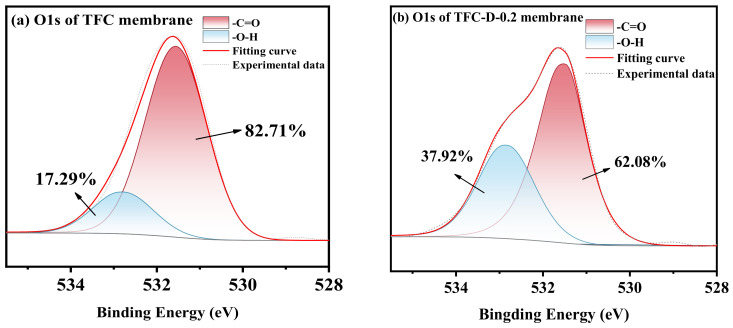
The O1s XPS spectra of the benchmark TFC (**a**) and TFC-D-0.2 (**b**).

The changes in chemical groups and elemental composition on the membrane surface were characterized using XPS. The wide scan spectrum is presented in the Supplementary Material Figure S2, and the deconvolution results of O1s are shown in Figure 2.

Peak-fitting analysis of the O1s XPS spectra (Figure 2) revealed that the intensity of the characteristic peak corresponding to hydroxyl groups (-OH) on the membrane surface was significantly enhanced after modification compared to the pristine membrane. This provides strong verification that D-glucamine molecules were successfully grafted onto the surface of the PA selective layer.

#### 3.1.2. Morphologies of OSRO Membranes

**Figure 3:**
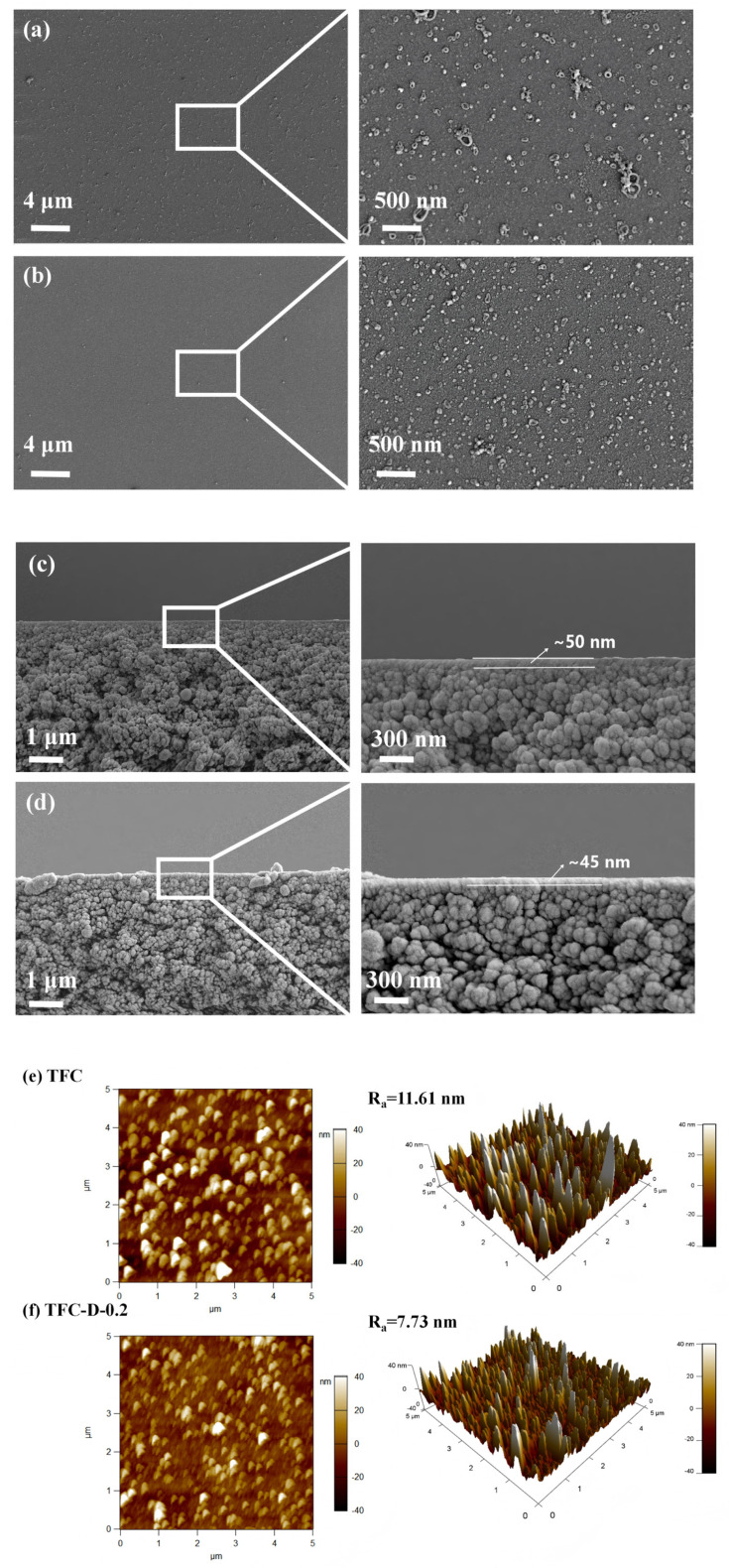
Surface SEM images of different membranes: (**a**) TFC, (**b**) TFC-D-0.2; SEM cross-section images of different membranes: (**c**) TFC membrane, (**d**) TFC-D-0.2 membrane; AFM images of different membranes: (**e**) TFC, (**f**) TFC-D-0.2.

The surface morphology and roughness of the OSRO membranes were characterized by SEM and AFM, and the results are shown in Figure 3.

Figure 3a–d shows the surface morphology of the TFC and the TFC-D-0.2 membranes. As can be seen from the figures, both membranes had a smooth, dense, and defect-free nodular structure. However, the nodular structure on the surface of the TFC-D-0.2 membrane was denser and more uniformly distributed than that of the pristine TFC membrane. It is known that after the IP reaction between MPD and TMC is complete, there still remain a large number of unreacted acyl chloride groups that have not reacted on the nascent PA membrane surface, either between the nodules or in certain areas. The amino groups (-NH_2_) of D-glucamine could react with the residual acyl chloride groups to form new amide bonds. These newly formed linkages enable the grafting of D-glucamine molecules on the membrane surface, thereby filling the originally loose regions or minor pores [33] and transforming the previously potentially heterogeneous structures into a more uniformly distributed and densely packed morphology [34,35]. Furthermore, ethanol exerts a certain swelling effect on the membrane surface, causing the originally rigidly coiled polymer chains to become extended after swelling [36,37]. Simultaneously, the numerous hydroxyl groups contained in D-glucamine form a hydrogen bond network on the membrane surface. This hydrogen bond network may pull the PA segments, bringing the originally dispersed segments closer together, thereby increasing the packing density of the surface layer [38]. The cross-sectional morphology further supports this conclusion. As shown in Figure 3c,d, after surface modification with D-glucamine, the thickness of the TFC-D-0.2 membrane skin layer decreased slightly from approximately 50 nm to about 45 nm. This could be attributed to the closer packing of PA segments induced by the hydrogen bond network, which leads to densification of the surface layer and a corresponding reduction in thickness [39]. Additionally, low-molecular-weight PA fragments may be dissolved by ethanol [40,41,42,43,44], which also contributes to the thinning of the skin layer.

Figure 3e,f shows the AFM images of the TFC and TFC-D-0.2 membranes, illustrating the surface roughness of the two membranes, respectively. As shown in Figure 3f and Table 3, after surface modification, the surface roughness of the TFC-D-0.2 membrane decreased from 11.6 nm to 7.7 nm. This result is consistent with the SEM surface morphology, indicating a more uniform nodular structure and a smoother membrane surface.

#### 3.1.3. Surface Hydrophilicity of the OSRO Membranes

**Figure 4:**
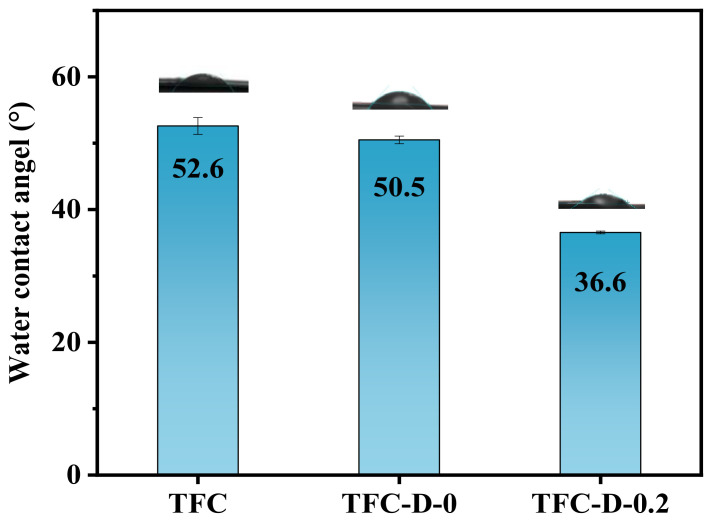
Water contact angle (WCA) of different membranes.

Figure 4 shows the water contact angle measurement of the TFC, TFC-D-0, and TFC-D-0.2 membranes. As shown in the figure, the water contact angle decreased gradually. The TFC-D-0.2 membrane exhibited the lowest water contact angle of 36.6°, indicating the strongest hydrophilicity. This can be primarily attributed to the introduction of abundant hydrophilic hydroxyl groups by D-glucamine, as confirmed by the XPS characterization. In addition, the swelling effect of ethanol on the membrane also facilitates the exposure of hydrophilic groups. Moreover, the use of ethanol prevents the hydrolysis of acyl chloride groups, thereby promoting the grafting reaction. Studies have shown that ethanol, as a non-solvent, can significantly increase the grafting rate of polymer membranes via the ethanol-induced membrane swelling and structural reorganization [45]. The combined effects significantly enhance the hydrophilic performance of the membrane surface.

### 3.2. Optimization of Surface Modification Conditions for the OSRO Membranes

**Figure 5:**
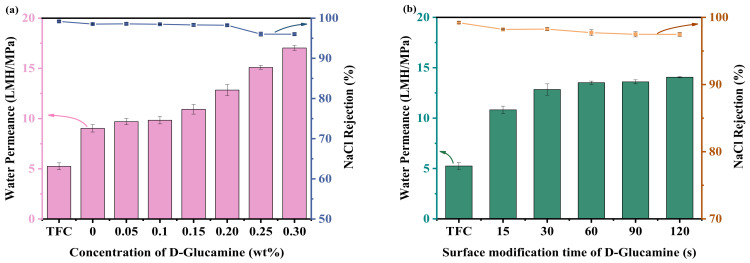
Effect of D-glucamine concentration (**a**) and surface modification time (**b**) on the separation per-formance of OSRO membrane, separation test with 2000 mg kg^−1^ NaCl aqueous solution as feed at 1.5 MPa and 25 ± 1 °C.

The effects of D-glucamine concentration and IP time on the separation performance of OSRO membranes were investigated, and the results are shown in Figure 5. It can be seen that the TFC membrane has a water permeance of only 5.25 LMH/MPa, but a much higher NaCl rejection of 99.20%. When the D-glucamine concentration was 0, i.e., the nascent PA membrane was treated by pure ethanol, the water permeance of the TFC-D-0 vigorously increased up to 9.03 LMH/MPa, significantly higher than that of the TFC membrane, accompanied by a slightly decreased NaCl rejection to 98.53%. This could be attributed to the swelling effect of ethanol on the membrane and the dissolution of low-molecular-weight PA fragments [40,41,46]. From a physical perspective, ethanol acts as a swelling agent that disrupts interchain hydrogen bonding within the polyamide matrix, increasing chain mobility and segmental freedom. This swelling effect allows for the rearrangement of polymer chains, leading to the formation of more open structures that facilitate water transport [47,48,49]. Simultaneously, the dissolution of unreacted or loosely bound oligomeric PA fragments creates additional pathways for solvent permeation, contributing to the observed flux enhancement. With the increase in the D-glucamine concentration and the extension of the surface modification time, a trend of continuously increasing water permeance and slightly decreasing NaCl rejection of the membranes was observed. With a D-glucamine concentration of 0.2 wt% and the surface modification time of 30 s, the water permeance reached 12.84 LMH/MPa, with a comparable NaCl rejection (~98.25%). With increasing concentration of the D-glucamine/ethanol solution, the water permeability of the membrane increased, while the NaCl rejection decreased, representing a typical trade-off effect. When the concentration of the D-glucamine/ethanol solution was 0.1 wt%, the water permeability of the TFC-D-0.1 membrane was 9.84 LMH/MPa, showing no significant improvement in permeation performance. When the concentration was increased to 0.3 wt%, the NaCl rejection of the TFC-D-0.3 membrane decreased to 96.01%. Given that a further increase in the concentration of the D-glucamine/ethanol solution was considered detrimental to membrane selectivity, TFC-D-0.2 was selected as the optimal membrane. The increase in the water permeance after D-glucamine modification could be due to the abundant hydroxyl groups of D-glucamine molecules. After being grafted onto the membrane surface, they significantly increase the surface hydrophilicity. The enhanced hydrophilicity, together with the reduced thickness of the selective layer, facilitates the adsorption of water molecules on the membrane surface and fosters their diffusion within the membrane, thereby reducing the water transport resistance and increasing the water permeance. Higher D-glucamine concentrations and longer modification times lead to more D-glucamine molecules being grafted, resulting in a greater density of surface hydroxyl groups and stronger hydrophilicity, hence the continuous increase in flux. However, when the concentration of D-glucamine was further increased (e.g., >0.2 wt%), the NaCl rejection slightly decreased. This phenomenon may be attributed to the excessive grafting of D-glucamine molecules, which could partially disrupt the dense PA network and increase the free volume of the selective layer. In addition, excessive grafting may cover part of the surface carboxyl groups and reduce the formation of hydrolysis-generated carboxyl groups [50,51]. Meanwhile, the abundant hydroxyl groups may form a stronger hydrogen-bonding network, which decreases the negative surface charge of the membrane and weakens the electrostatic repulsion toward salt ions, thereby leading to a decline in NaCl rejection [52,53]. As the modification time increases, the swelling effect of ethanol accumulates, and the membrane structure gradually loosens, resulting in an increase in flux and a decrease in rejection.

### 3.3. Separation Performance of OSRO Membranes

**Figure 6:**
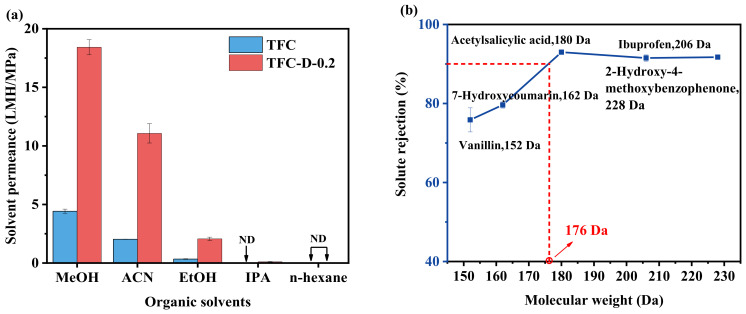
(**a**) Pure solvent flux of the TFC and TFC-D-0.2 membrane, and (**b**) MWCO of the TFC-D-0.2 membrane.

The pure solvent permeability performance of the TFC and TFC-D-0.2 membranes was evaluated using five different solvents. As shown in Figure 6a, for both membranes, the permeance followed the order: methanol > acetonitrile > ethanol > isopropanol ≈ n-hexane. The difference in permeance could be attributed partially to the affinity between the solvent and the PA separation layer, and partially to the properties of the solvents. The affinity between the solvent and the PA separation layer can be expressed using the Hansen solubility parameters (*HSP*). According to the principle of “like dissolves like” [54,55], a smaller solubility parameter distance (*S_a_*) between the two substances indicates stronger affinity. The Hansen solubility parameters and the *S_a_* are calculated using the following equations [56]:

(3) $$\mathrm{HSP}=\sqrt{\left({\delta_{d}}^{2}+{\delta_{p}}^{2}+{\delta_{h}}^{2}\right)}$$

(4) $$S_{a}=\sqrt{\left[4{\left(\delta_{d,m}-\delta_{d,s}\right)}^{2}+{\left(\delta_{p,m}-\delta_{p,s}\right)}^{2}+{\left(\delta_{h,m}-\delta_{h,s}\right)}^{2}\right]}$$

Among them, $\delta_{d}$, $\delta_{p}$ and $\delta_{h}$ are the dispersion, polar, and hydrogen-bonding components of the HSP, respectively, and the subscripts m and s refer to the membrane and solvent, respectively. Accordingly, $\delta_{d,m}$ and $\delta_{d,s}$ represent the dispersion components of the HSP for the membrane and the solvent, respectively. $\delta_{p,m}$ and $\delta_{p,s}$ represent the polar components. $\delta_{h,m}$ and $\delta_{h,s}$ represent the hydrogen-bonding components.

The molecular weight, viscosity, molar volume, molecular diameter, and Hansen solubility parameters of the five pure solvents used in this experiment, as well as the distance between Hansen solubility parameters of solvent and polyamide layer, are shown in Table 4.

It can be seen from Table 4 that the order of *S_a_* is: acetonitrile < isopropanol < ethanol < methanol ≈ n-hexane, indicating that the membrane exhibits the strongest affinity with acetonitrile, followed by isopropanol, and the weakest affinity with methanol. Generally, solvents with a stronger affinity are believed to be more likely to dissolve and diffuse in the membrane, thus achieving higher permeability. However, contrary to this expectation, methanol, which exhibited the weakest affinity, displayed the highest permeability, with permeabilities of 4.42 LMH/MPa and 16.58 LMH/MPa in the TFC and TFC-D-0.2 membranes, while isopropanol, which has a stronger affinity, had the lowest permeability. Further analysis revealed that the permeabilities of the four solvents excluding n-hexane were negatively correlated with their molecular size. For example, methanol, which has the smallest molecular size (Table 4), had the highest permeability, whereas isopropanol, with a larger molecular size, exhibited the lowest permeability. This indicates that the size of the solvent plays a decisive role in this separation system. Additionally, solvent viscosity also influences permeation performance, with the permeation order generally consistent with the viscosity order except for methanol. This indicated that solvents with lower viscosity are more easily transported through the membrane. Acetonitrile, due to its relatively small molecular diameter (molar volume) and the lowest viscosity, exhibited a high mass permeation flux in both membranes. However, the molecular diameter and viscosity of ethanol are both higher than those of methanol and acetonitrile, resulting in a significantly reduced permeance of only 0.34 LMH/MPa and 1.86 LMH/MPa in the TFC and TFC-D-0.2 membranes. The viscosity of isopropanol is approximately twice that of ethanol, as shown in Table 4. If mass transfer were solely governed by viscosity, the flux of isopropanol should be roughly half that of ethanol. However, the experimental results showed that the permeation flux of isopropanol was nearly zero for both membranes. This significant deviation can only be attributed to the size sieving effect: the kinetic diameter of isopropanol (approximately 0.62 nm) likely approaches or exceeds the pore size of the membrane’s selective layer, hindering its transport. Based on the permeance behavior of isopropanol, it can be inferred from this that the average pore size of the prepared OSRO membranes is comparable to the molecular diameter of isopropanol. For the non-polar solvent n-hexane, although its viscosity is the lowest among all the solvents tested, its molecular diameter (0.75 nm) is the largest. This size significantly exceeds the membrane pore size inferred from the permeation behavior of isopropanol. Consequently, n-hexane molecules are completely blocked sterically, resulting in nearly zero permeation flux for both the TFC and TFC-D-0.2 membranes. In addition, the difference in Hansen solubility parameters between the selective layer and n-hexane is relatively large, indicating poor affinity between the membrane material and hexane. Such unfavorable compatibility reduces the sorption of hexane molecules within the membrane matrix, thereby hindering their diffusion through the selective layer and resulting in the negligible permeability.

To determine the MWCO of the TFC-D-0.2 membrane, separation performance tests were conducted using five different mixtures of small organic molecules/ethanol mixtures with varying molecular weights, and the results are shown in Figure 6b. It can be observed that the rejection of solutes by the TFC-D-0.2 membrane exhibited a clear increasing trend with increasing molecular weight. Specifically, the rejection of vanillin (152 Da) and 7-hydroxycoumarin (162 Da) was 75.9% and 79.6%, respectively, both below 90%. However, when the molecular weight increased to 180 Da (acetylsalicylic acid) and above, the rejection consistently exceeded 90%. The rejection for acetylsalicylic acid (180 Da), ibuprofen (206 Da), and 2-hydroxy-4-methoxybenzophenone (228 Da) reached 92.9%, 91.5%, and 91.7%, respectively. Based on this trend, it can be inferred that the MWCO of the TFC-D-0.2 membrane is approximately 176 Da.

### 3.4. Stability Performance of the OSRO Membranes

**Figure 7:**
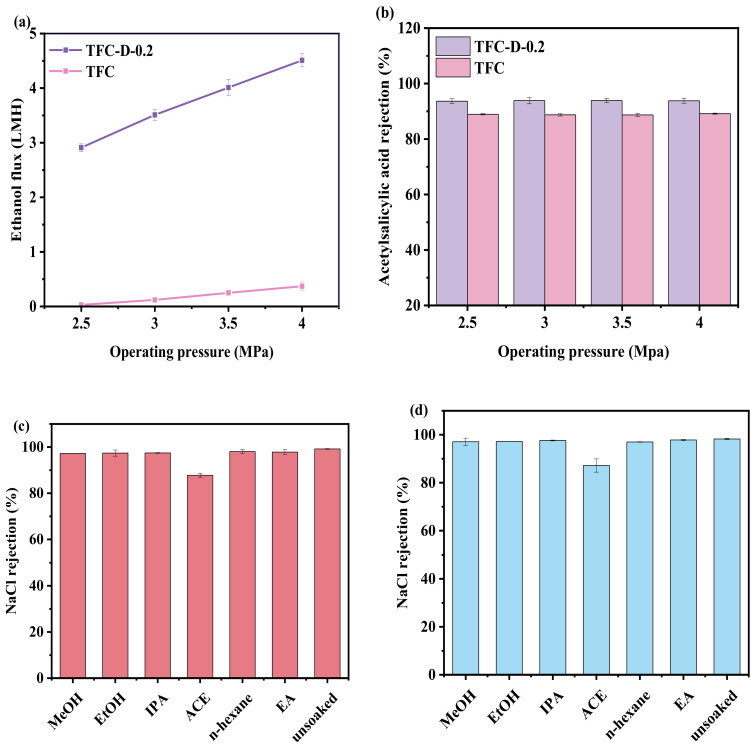
Effect of operating pressure on ethanol flux (**a**) and acetylsalicylic acid rejection (**b**) of TFC-D-0.2 and TFC membranes; Solvent resistance of TFC membrane (**c**) and TFC-D-0.2 membrane (**d**) after static immersion in different solvents for 30 days, separation test with 2000 mg kg^−1^ NaCl aqueous solution as feed at 1.5 MPa and 25 ± 1 °C.

In order to evaluate the operational stability of the membranes, their performance was investigated from two aspects: pressure and solvent resistance. The pressure resistance of the TFC and TFC-D-0.2 membranes was tested with a 100 mg kg^−1^ acetylsalicylic acid/ethanol solution. As shown in Figure 7a,b, increasing the operating pressure increased the ethanol flux while having little effect on the rejection of acetylsalicylic acid. As the operating pressure increased from 2.5 MPa to 4.0 MPa, the ethanol flux of both membranes exhibited a linear increasing trend. Specifically, the ethanol flux of the TFC-D-0.2 membrane increased from 2.91 LMH to 4.51 LMH, while its rejection for acetylsalicylic acid remained stably above 93.5%. Meanwhile, the ethanol flux of the TFC membrane increased from 0.03 LMH to 0.37 LMH, and its acetylsalicylic acid rejection remained above 88.50%. These results indicate that within the pressure range of 2.5–4.0 MPa, both the TFC and TFC-D-0.2 membranes maintained good separation performance, with no noticeable decline in rejection observed as the pressure increased, confirming their excellent pressure resistance. This linear relationship between flux and pressure is consistent with the solution-diffusion model [58], where the solvent flux is proportional to the applied pressure gradient. The absence of rejection decline with increasing pressure provides important physical insight into the membrane’s structural integrity. The stable rejection across the pressure range indicates that the polyamide network, reinforced by the D-glucamine modification, maintains its structural integrity and size-sieving capability under hydraulic stress. This mechanical robustness can be attributed to the hydrogen-bonding network formed among the grafted D-glucamine molecules, which acts as a physical crosslinking mechanism that stabilizes the polyamide chains against pressure-induced deformation. It is worth mentioning that the ethanol permeance of the TFC-D-0.2 membrane in this study is comparable to that reported by Matsuyama et al. [13], which was approximately 1.9 LMH at an operating pressure of 7.0 MPa, corresponding to 0.27 LMH/MPa. At 4 MPa, the ethanol flux of the TFC-D-0.2 membrane is approximately 12 times that of the TFC membrane. This enhancement can be attributed to the increased hydrophilicity of the membrane surface, as evidenced by the WCA results. Meanwhile, the swelling effect of ethanol on the membrane may also contribute to the improved permeance, as demonstrated in Figure 5.

To assess the organic solvents’ resistance, both OSRO membranes were immersed in six solvents (methanol, ethanol, isopropanol, acetone, n-hexane, and ethyl acetate) at room temperature for 30 days, and the results are shown in Figure 7c,d. Compared to the un-soaked membranes, the most significant decrease in rejection for both membranes was observed after immersion in acetone. The NaCl rejection of the TFC membrane dropped from 99.20% to 87.79%, while that of the TFC-D-0.2 membrane decreased from 98.25% to 87.18%. This phenomenon can be attributed to the small Hansen solubility parameter distance between acetone and the membrane material [59]. According to the principle of “like dissolves like” [54,55], a smaller solubility parameter distance indicates greater affinity. Acetone can easily diffuse into the membrane and cause significant swelling, leading to loosening of the PA network structure and enlargement of ion transport channels [60]. In contrast, after immersion in the other solvents, the NaCl rejection of the TFC membrane remained above 97%, and that of the TFC-D-0.2 membrane remained above 96.50%, indicating that these solvents caused minimal damage to the membrane structure.

### 3.5. Industrial Application Potential of OSRO Membranes

**Figure 8:**
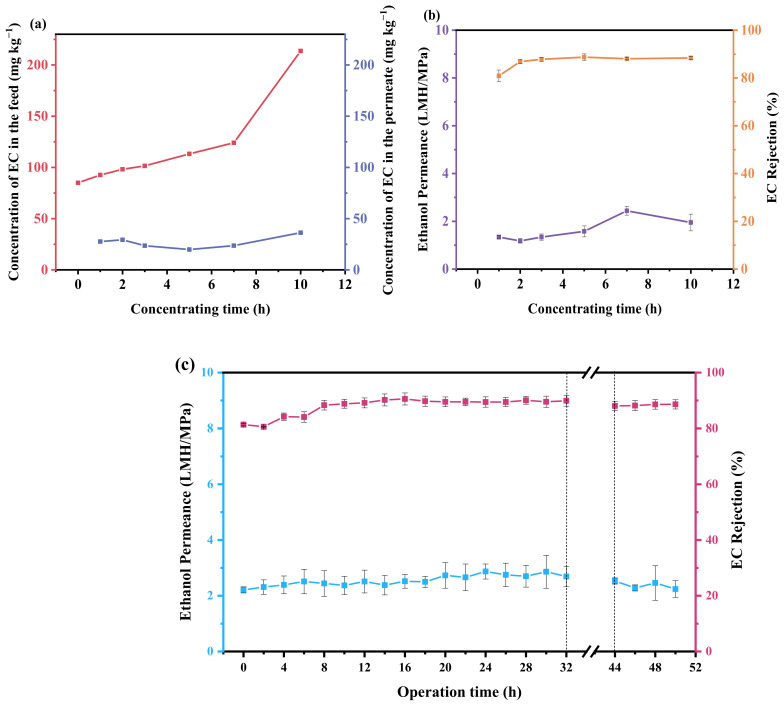
(**a**) The change in the ethyl cinnamate content in the feed and the permeate solutions and (**b**) The filtration performance of the TFC-D-0.2 membrane for ethyl cinnamate/ethanol solution and (**c**) Long-term operational stability of the TFC-D-0.2 membrane with 100 mg/kg EC/ethanol solution as feed at 3.0 MPa and 25 ± 1 °C (The breakpoint indicates a period of static contact with the same solution).

Ethyl cinnamate is an important functional compound with considerable value in a variety of industrial sectors, particularly in fine chemicals. It is commonly synthesized through the esterification reaction of cinnamic acid with ethanol, catalyzed by sulfuric acid. This process yields a mixture of ethyl cinnamate and ethanol, with a yield of approximately 60%. To test the separation performance of the membrane for ethyl cinnamate/ethanol mixtures, the TFC-D-0.2 membrane was employed to concentrate an 85 mg kg^−1^ ethyl cinnamate/ethanol solution, and the results are shown in Figure 8. As can be seen from Figure 8a, during the 10 h concentration process, the concentration of ethyl cinnamate in the feed solution continuously increased with concentration time, while the concentration of ethyl cinnamate in the permeate remained at a relatively stable level. In the final 3 h, the concentration of ethyl cinnamate in the feed solution increased significantly. Ultimately, the ethyl cinnamate/ethanol mixture was concentrated by more than twofold, and the performance of the TFC-D-0.2 membrane remained stable during the 10 h operation, with ethanol permeance maintaining around 2 LMH/MPa and the ethyl cinnamate rejection at approximately 85%.

As shown in Figure 8c, the long-term stability of the TFC-D-0.2 membrane was tested using a 100 mg·kg^−1^ ethyl cinnamate/ethanol solution at 3.0 MPa. During the first 8 h of operation, there was a gradual increase in ethanol permeance, which increased from 2.21 LMH/MPa to 2.44 LMH/MPa, and the ethyl cinnamate rejection gradually increased from 81.33% to 88.32%. The increase in ethanol permeance can be attributed, on the one hand, to the initial stage of high-pressure operation where the membrane contacts ethanol, gradually opening up originally coiled or incompletely opened nanochannels [61], forming pathways more conducive to solvent transport. This reduces the trans-membrane resistance for ethanol molecules. Coupled with the swelling effect of ethanol [60], the synergistic action of these factors ultimately leads to an increase in membrane permeance. The increase in ethyl cinnamate rejection may be attributed to the gradual uncoiling and rearrangement of polymer segments during the ethanol swelling process. After 8 h of operation, the ethanol permeance of the membrane gradually stabilized at 2.7 LMH/MPa, while the ethyl cinnamate rejection remained around 89%. Following 50 h of long-term operation, the ethanol permeance of the TFC-D-0.2 membrane remained at approximately 2.5 LMH/MPa, and the ethyl cinnamate rejection remained above 88%, demonstrating that the membrane surface was not damaged and remained intact. This indicates that the TFC-D-0.2 membrane possesses good long-term operational stability. Although no specialized mechanical tests were conducted, the stable performance during long-term filtration experiments indirectly verifies the mechanical reliability of the membrane.

### 3.6. Compared with Other OSRO Membranes Reported in the Literature

The performance of the TFC-D-0.2 membrane was compared with state-of-the-art hydrophilic OSRO membranes fabricated via interfacial polymerization in recent years, as summarized in Table 5. The TFC-D-0.2 membrane exhibits excellent permeance while maintaining high selectivity, with a methanol permeance of 16.58 LMH/MPa and water permeance of 12.84 LMH/MPa combined with 98.25% NaCl rejection, ranking among the highest values. Compared to membranes requiring complex fabrication strategies such as carbon nanotube interlayers (PK-CNTs-PA) [14] or fluorine incorporation (F-PA) [11], our approach utilizes a simple, cost-effective surface modification with D-glucamine, an environmentally benign amino sugar alcohol, achieving a well-balanced trade-off between permeability and selectivity. Furthermore, the TFC-D-0.2 membrane demonstrates robust long-term stability, maintaining stable performance after 30 days of immersion in most organic solvents and during 50 h of continuous operation. Overall, the combination of high permeance, good selectivity, simple fabrication, and excellent stability positions the TFC-D-0.2 membrane as a promising candidate for green organic solvent separation in fine chemical industries.

## 4. Conclusions

In summary, this study successfully prepared a high-performance OSRO membrane (TFC-D-0.2) via D-glucamine surface modification, demonstrating excellent hydrophilicity, solvent resistance, and potential for fine chemical purification. The optimal membrane (TFC-D-0.2) exhibited an acetylsalicylic acid (180 Da) rejection of 93.8% and an ethanol permeance of 1.87 LMH/MPa, and maintained NaCl rejection above 96.50% after 30 days of immersion in most ordinary solvents. Although several limitations remain—notably performance degradation in acetone due to strong swelling and the lack of long-term stability evaluation beyond 50 h under mild conditions—this study provides a new strategy for developing high-performance OSRO membranes and lays a foundation for the green advancement of organic solvent mixture separation technology.

## Figures and Tables

**Table 1:** Membrane fabrication conditions.

Type of Membrane	Aqueous Phase	Organic Phase	Surface Modification
	MPD (wt%)	TMC (wt%)	D-glucamine (wt%)
TFC	2.0	0.15	/
TFC-D-0	2.0	0.15	0
TFC-D-0.1	2.0	0.15	0.1
TFC-D-0.2	2.0	0.15	0.2
TFC-D-0.3	2.0	0.15	0.3
TFC-D-0.4	2.0	0.15	0.4

**Table 2:** The chemical structures, molecular weights, and molecular dimensions of the five small-molecule compounds.

**Chemica**	**Molecular** **Weight (Da)**	**Molecular Structure**	**3D Molecular Structure**	**Dimension (nm) (L1 × L2 ×** **L3** **, L1 is the Long End** **,** **L2 is the Middle End** **and** **L** **3** **is the Short End) ^a^**
Vanillin	152	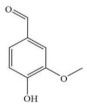	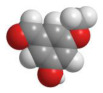	0.70 × 0.52 × 0.18
7-hydroxycoumarin	162	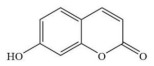	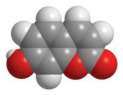	0.74 × 0.50 × 0.11
Acetylsalicylic acid	180	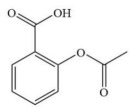	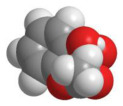	0.76 × 0.50 × 0.39
Ibuprofen	206	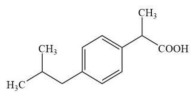	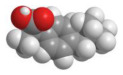	0.11 × 0.50 × 0.43
2-hydroxy-4-methoxybenzophenone	228	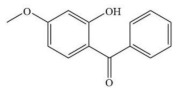	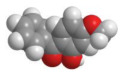	0.11 × 0.56 × 0.50

^a^ The 3D molecular structure and size are calculated using ChemDraw 22.0.0.22 software.

**Table 3:** Roughness and normalized area of the OSRO membrane.

Membrane	R*_a_*(nm)	R*_q_*(nm)	Area (μm^2^)
TFC	11.6	14.9	25.9
TFC-D-0.2	7.7	10.4	25.7

**Table 4:** Properties and Hansen solubility parameter of the used solvents and the PA membrane in this experiment [12,57].

Solvent and Membrane	Molecular Weight (Da)	Molecular Diameter (nm)	Molar Volume (cm^3^/mol)	Viscosity (cP)	*δ*_d_ (MPa^1/2^)	*δ*_p_ (MPa^1/2^)	*δ*_h_ (MPa^1/2^)	*HSP* (MPa^1/2^)	*S*_a_ (MPa^1/2^)
MeOH	32	0.51	40.7	0.54	15.1	12.3	22.3	29.6	15.5
ACN	41	0.55	52.6	0.37	15.3	18.0	6.1	24.4	8.3
EtOH	46	0.57	58.5	1.07	15.8	8.8	19.4	26.5	12.7
IPA	60	0.62	76.8	2.04	15.8	6.1	16.4	23.6	11.2
Acetone	58	0.62	75.1	0.32	15.5	10.4	7.0	19.9	5.3
n-hexane	86	0.75	131.6	0.29	14.9	0	0	14.9	15.6
EA	88	0.67	98.0	0.45	15.8	5.3	7.2	18.1	8.0
polyamide	—	—	—	—	18.0	11.9	7.9	23.0	—

**Table 5:** Performance comparison of the TFC-D-0.2 membrane with state-of-the-art hydrophilic OSRO membranes reported in the literature.

Membranes	Pure Solvent Permeance		Separation Performance ^a^		Reference and Year
	Solvent	Permeance (LMH/MPa)	Solvent Permeance (LMH/MPa)	Solute Rejection (%)	
TFC-D-0.2	MeOH	16.58	Water, 12.58	NaCl, 98.25	This work
	ACN	9.96	EtOH, 3.51	Acetylsalicylic acid, 93.85	
	EtOH	1.87			
PK-RO	MeOH	4.70	Water, 5.00	NaCl, 97	2020 [6]
	Acetone	1.10			
	EtOH	0.50			
A130W95 PK	MeOH	3.50	Water, 6.00	NaCl, 99	2021 [7]
	ACN	2.30			
	EtOH	0.41			
F-PA	MeOH	12.80	Water, /	NaCl, 98.3	2022 [11]
	EtOH	10.10	Toluene, 1.40	TIPB, 93.9	
PA-TFC-PK-70	MeOH	6.00	Water, 4.5	NaCl, >98	2023 [9]
PA-TFC-PK-90	MeOH	5.70	Water, 3.5	NaCl, >98	2023 [9]
PK-CNTs-PA	MeOH	13.90	Water, 13.2	NaCl, 98.4	2023 [14]
	ACN	21.30	MeOH, 2.10	MTBE, 98.0	
	EtOH	3.10			
PA-PK6	MeOH	12.30	Water, 5.60	NaCl, 98	2023 [8]
	ACN	13.50	MeOH, 4.71	MTBE, 90.0	
	EtOH	3.40			
PA-Fe^3+^/TA P84	MeOH	3.20	/	/	2024 [12]
	ACN	2.80			
	EtOH	0.30			
TFC-PA-CS-0.2	MeOH	3.41	/	/	2025 [13]
	EtOH	3.17			
TFC-PA	MeOH	1.23	/	/	2025 [13]
	EtOH	1.90			
PEI-TMC PK	MeOH	15.00	MeOH, 6.82	Hexane, 97.5	2025 [10]
	ACN	12.00	MeOH, 7.15	MTBE, 98.0	
	EtOH	3.00			
MPD-TMC PK	MeOH	8.00	MeOH, 3.60	Hexane, 97.8	2025 [10]
	ACN	4.80	MeOH, 3.86	MTBE, 98.8	
	EtOH	1.40			

^a^: Separation performance refers to the solvent permeance and solute rejection corresponding to the solutions of different systems in the table below.

## Data Availability

The original contributions presented in this study are included in the article and Supplementary Material. Further inquiries can be directed to the corresponding author.

## Supplementary Materials

The following supporting information can be downloaded at: https://www.mdpi.com/article/10.3390/membranes16050171/s1, Figure S1: Absorbance curves for (a) different vanillin concentrations in ethanol solution at 231.0 nm, (b) different 7-hydroxycoumarin concentrations in ethanol solution at 205.0 nm, (c) different acetylsalicylic acid concentrations in ethanol solution at 224.0 nm, (d) different ibuprofen concentrations in ethanol solution at 217.0 nm, (e) different 2-hydroxy-4-methoxybenzophenone concentrations in ethanol solution at 387 nm, (f) different ethyl cinnamate concentrations in ethanol solution at 23.0 nm; Figure S2: The wide scan spectrum of TFC and TFC-D-0.2; Figure S3: XPS spectra: (a, c) C1s, N1s images of the TFC membrane, (b, d,) C1s, N1s images of the TFC-D-0.2 membrane; Figure S4: Effect of MPD concentration (a), TMC concentration (b), aqueous phase immersion time (c), and organic phase immersion time (d) on the separation performance of OSRO membranes; Figure S5: Effect of heat treatment on OSRO membrane separation performance; Figure S6: SEM cross-section images of different membranes: (a) TFC membrane, (b) TFC-D-0.2 membrane; Figure S7: WCA of 5 types of membranes.
