# Supplementary material for: High-Flux Solvent-Resistant Reverse Osmosis Membrane Enabled by D-glucamine Surface Modification
Source: Membranes (Basel). 2026 May 6;16(5):171. doi: 10.3390/membranes16050171 (PMC13208494; doi:10.3390/membranes16050171)
Supplement: Supplementary file 1 [file membranes-16-00171-s001.zip › membranes-4219314-supplementary.pdf]

Supplementary materials

High-flux solvent-resistant reverse osmosis membrane enabled by D-glucamine surface modification

Bing Wang[a],Weijia Song[a], Yuqi Sun[a], Enlin Wang[a], Can Li[b,], Baowei Su[a,*]

[a] Key Laboratory of Marine Chemistry Theory and Technology (Ocean University of China), Ministry of Education/ College of Chemistry & Chemical Engineering, Ocean University of China, 238 Songling Road, Qingdao, 266100, China

[b] Singapore Membrane Technology Centre, Nanyang Environment and Water Research Institute, Nanyang Technological University, 1 Cleantech Loop, 637141, Singapore

* Correspondence: subaowei@ouc.edu.cn

S1. Concentration-absorbance curves of several small molecule substances

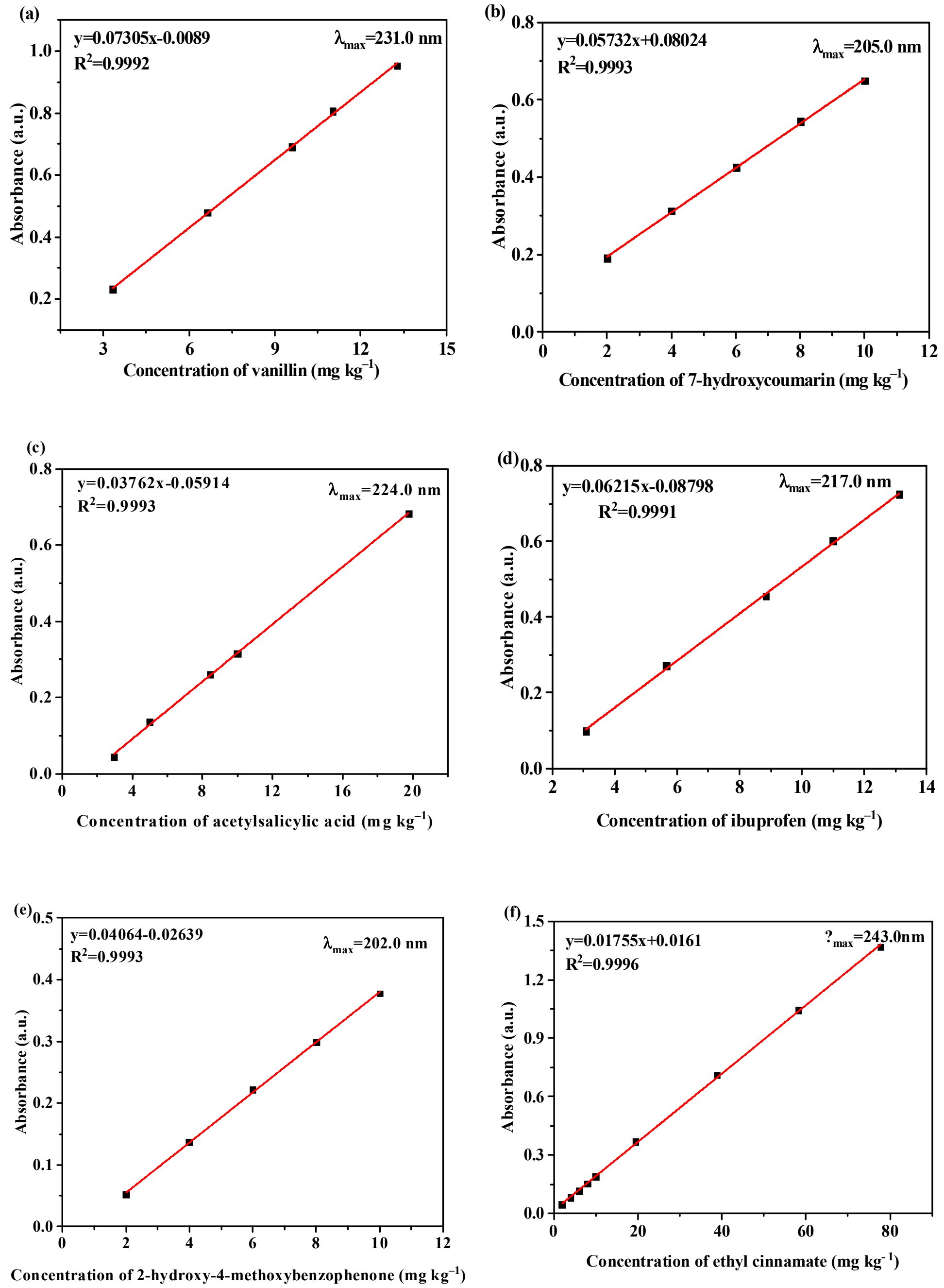

Fig. S1 Absorbance curves for (a) different vanillin concentrations in ethanol solution at 231.0 nm, (b) different 7-hydroxycoumarin concentrations in ethanol solution at 205.0 nm, (c) different acetylsalicylic acid concentrations in ethanol solution at 224.0 nm, (d) different ibuprofen concentrations in ethanol solution at 217.0 nm, (e)

different 2-hydroxy-4-methoxybenzophenone concentrations in ethanol solution at 387 nm, (f) different ethyl cinnamate concentrations in ethanol solution at 23.0 nm

S2. XPS wide scan spectrum of two types of membranes

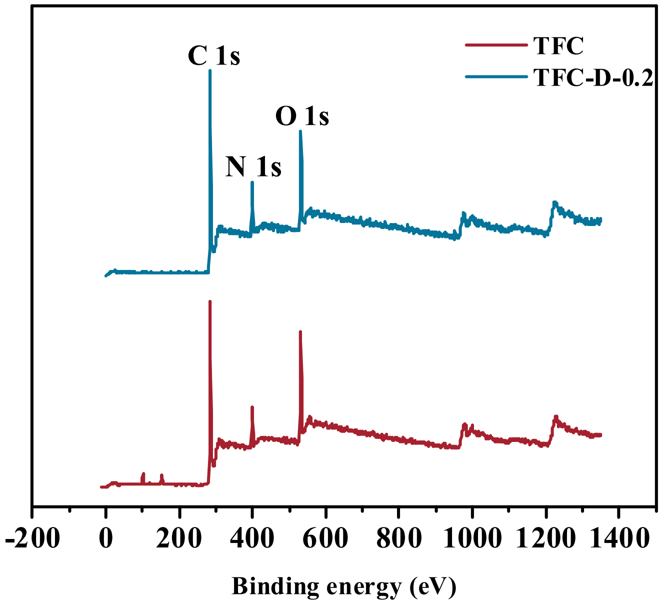

Fig. S2 The wide scan spectrum of TFC and TFC-D-0.2

S3. Peak fitting of the C1s and N1s spectra for the two membranes.

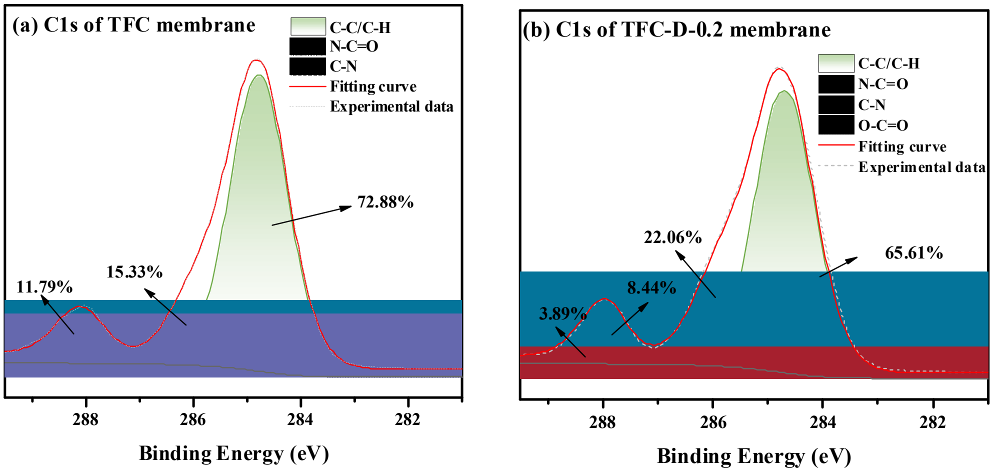

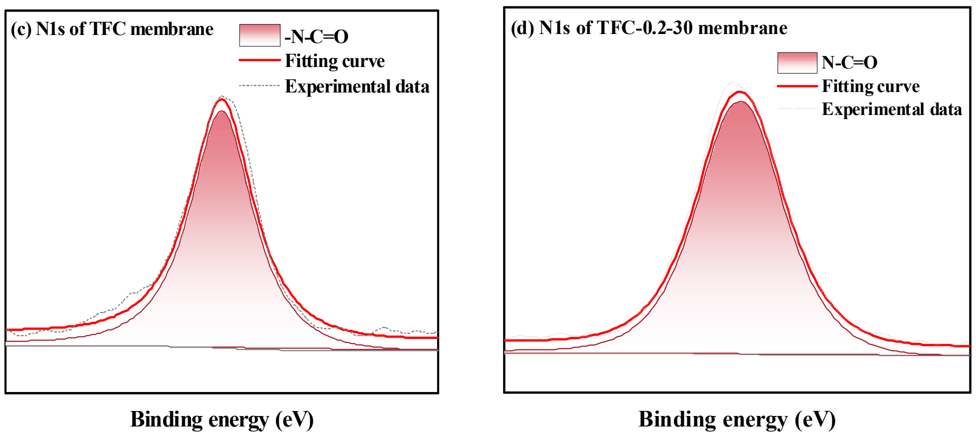

Fig. S3 XPS spectra: (a, c) C1s, N1s images of the TFC membrane, (b, d,) C1s, N1s images of the TFC-D-0.2 membrane

S4. Optimization of the Benchmark Membrane

The effects of MPD concentration, TMC concentration, aqueous phase immersion time, and organic phase immersion time on the separation performance of the benchmark TFC OSRO membrane were investigated. The final determined parameters were: MPD concentration of 0.2 wt%, TMC concentration of 0.2 wt%, aqueous phase immersion time of 16 s, and organic phase immersion time of 12 s.

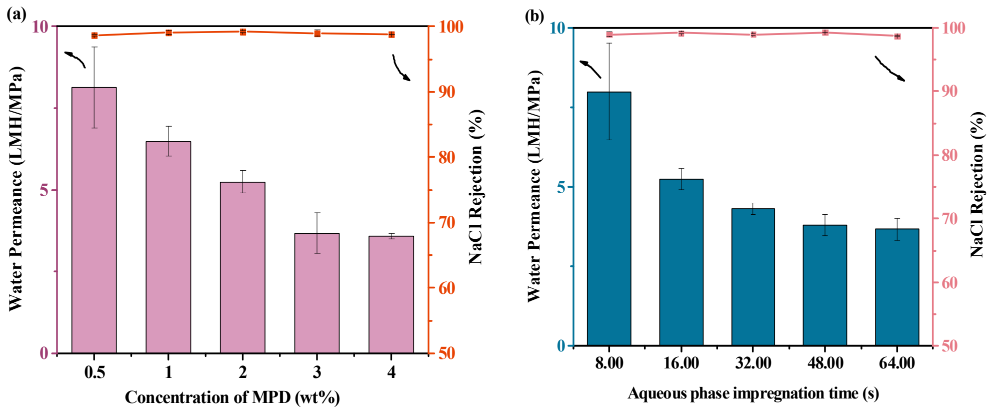

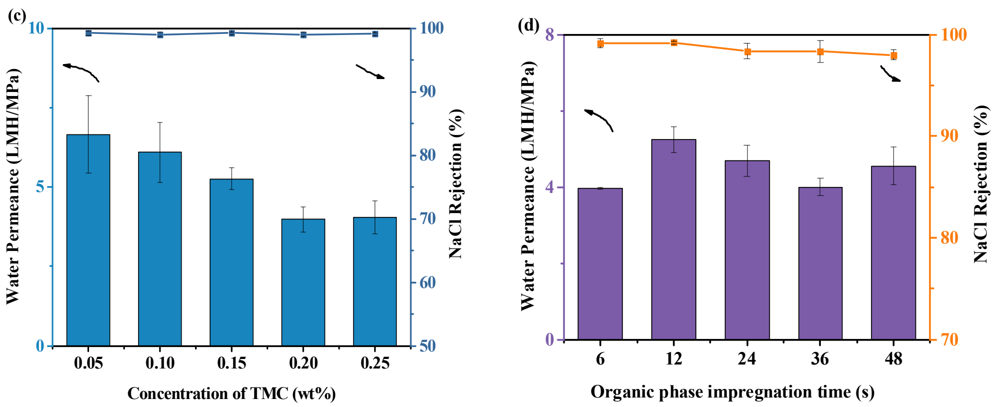

Fig. S4 Effect of MPD concentration (a), TMC concentration (b), aqueous phase immersion time (c), and organic phase immersion time (d) on the separation performance of OSRO membranes

S5. Effect of Heat Treatment on Membrane Separation Performance

Based on the experimental results regarding whether the three types of membranes were subjected to heat treatment, the membrane preparation condition was determined to be no heat treatment.

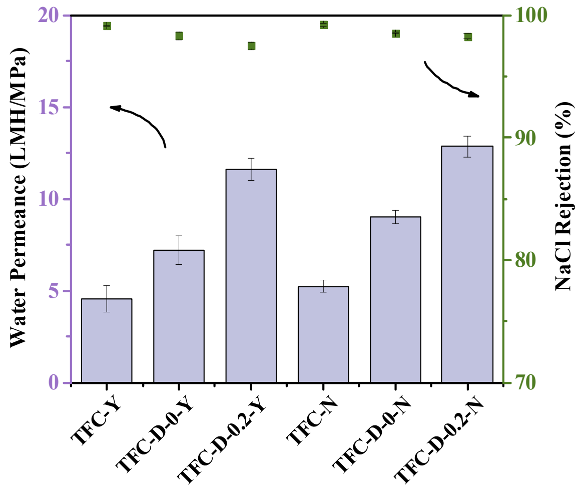

Fig. S5 Effect of heat treatment on OSRO membrane separation performance

S6. SEM cross-section images of different membranes

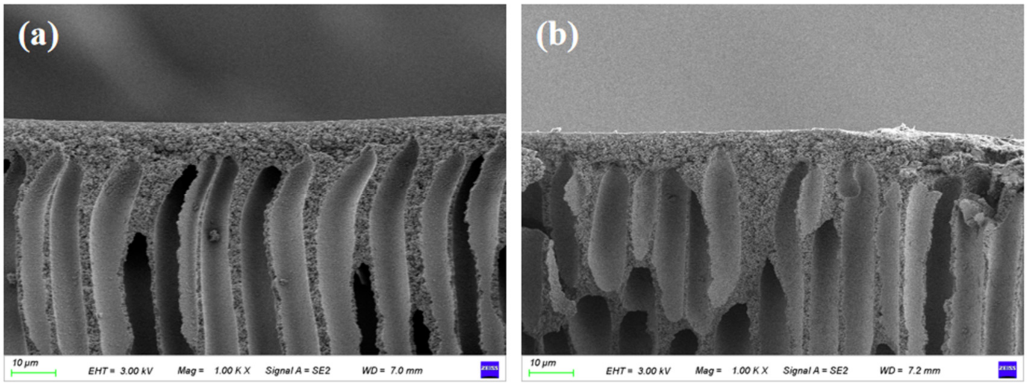

Fig. S6 SEM cross-section images of different membranes: (a) TFC membrane, (b) TFC-D-0.2 membrane

S7. WCA of different membranes

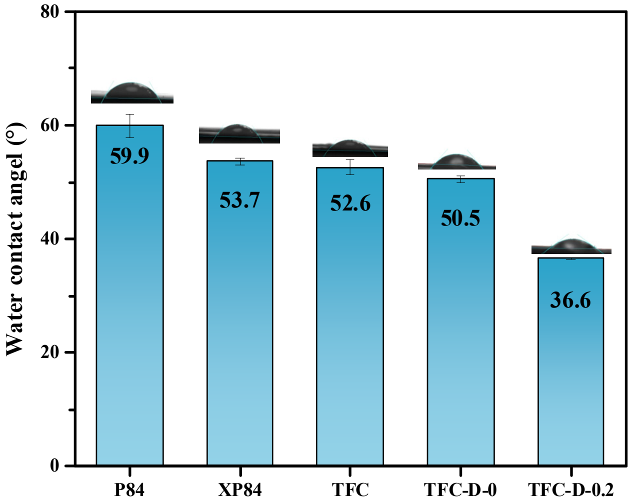

Fig. S7 WCA of 5 types of membranes

As shown in **Fig. S7**, P84 represents the PI ultrafiltration support membrane, and XP84 represents the crosslinked PI ultrafiltration support membrane.
